# Pharmacologic modulation of orexin signaling: Impact on glucose–insulin homeostasis and potential mechanism of antidepressant action

**DOI:** 10.1017/S1092852926101126

**Published:** 2026-07-29

**Authors:** Roger S. McIntyre, Sabrina Wong

**Affiliations:** 1 Department of Psychiatry, University of Toronto, Canada; 2 University Health Network, Canada; 3 Pharmacology & Toxicology, University of Toronto, Canada

**Keywords:** Seltorexant, suvorexant, lemborexant, daridorexant, orexin, depression, metabolism, antidepressant, DORA, SORA

## Abstract

In addition to their canonical role in sleep–wake regulation, endogenous orexin signaling is implicated in the homeostatic control of reward and motivation, stress and arousal, and glucose–insulin metabolism. Recent research has focused on the development of orexin antagonists, notably seltorexant, as adjunctive treatments in adults with major depressive disorder (MDD). Converging evidence implicates insulin resistance in MDD pathophysiology, highlighting the need to integrate preclinical, pharmacologic, and translational evidence examining the effects of orexin signaling on glucose–insulin homeostasis. A literature review was conducted using PubMed from database inception to June 2025 to identify preclinical and clinical studies examining the metabolic effects of orexin modulation. The search string included the terms related to: orexin, glucose, metabolism, and depression. Results were synthesized qualitatively across experimental models and clinical populations. Evidence indicates that orexin signaling affects multiple aspects of metabolic homeostasis. For example, orexins affect appetitive/consummatory behavior, exert incretin-like effects on insulin release and glucagon suppression, and increase energy expenditure via morphologically transitioning white-to-brown adipose tissue. These effects are mediated through both direct (eg, inhibition of gluconeogenic enzyme expression) and indirect pathways (eg, alterations in sleep architecture and attenuation of sympathoadrenal overactivity). Orexin antagonism, therefore, exerts multi-faceted physiological effects beyond sleep regulation. The antidepressant efficacy of seltorexant in MDD is greater in persons with moderate-to-severe insomnia, while remaining clinically meaningful in persons with less severe insomnia, implicating multiple mechanisms. Modulation of glucose–insulin homeostasis represents a putative, non-mutually exclusive mechanism through which orexin antagonism may contribute to antidepressant effects.

## Introduction

Orexins, also referred to as hypocretins, were discovered in 1998 and are a product of the prepro-orexin gene.^1^ The hypocretin designation (ie, “hypothalamic incretins”) was proposed for orexins due to the structural and functional similarity between them and incretins produced by the K (ie, glucose–insulinotropic polypeptide) and L (glucagon-like peptide-1; GLP) cells of the gastrointestinal tract.^2^ Two orexin proteins have since been identified, orexin 1 and orexin 2 (also known as hypocretin-1 and -2, respectively). The discovery of the orexin proteins de-orphanized the previously characterized G-protein–coupled orexin receptors (GPCRs), which are post-synaptic and are usually characterized as excitatory.^3^ In addition, orexin 1 has similar affinity for the orexin 1 (OX1R) and orexin 2 receptors (OX2R), while orexin 2 has relatively greater affinity for the OX2R.^2^

Soon after the discovery of orexins, their integral role in sleep–wake behavior was recognized.^4^ For example, discovery of functional gene variants for the OX2R, as well as for the orexin proteins, underscored the pathophysiological role of orexin deficiency in animal models of narcolepsy.^4^ ^,^^5^ Subsequently, it was discovered that loss of orexin-containing neuron function in humans was the mechanistic basis of type 1 narcolepsy (ie, with cataplexy).^4^ Consequent to the discovery of the orexins and their functional role in sleep–wake disturbance, three dual-orexin receptor antagonists (ie, DORAs; suvorexant, lemborexant, and daridorexant) were FDA-approved for the treatment of chronic insomnia in adults.

In addition to targeting sleep–wake homeostasis, centrally produced orexins, via their projections to cortical and subcortical structures, were discovered to govern energy expenditure, metabolism, reward/consummatory behavior as well as cognitive functions (eg, memory consolidation).^1^ Neuroanatomically, orexin-synthesizing neurons are located in the perifornical area (PFA) and lateral hypothalamus (LH). Projections from the perifornical neurons to the brainstem and other hypothalamic nuclei are responsible for arousal and vigilance.^6^ In contrast, projections from the LH to the ventral tegmental area (VTA) and ventral prefrontal medial cortex modulate reward, reinforcement, and motivational behavior.^7^ Epidemiological evidence indicates that adults with type 1 narcolepsy are differentially affected by metabolic dysregulation, including obesity, type 2 diabetes mellitus (T2DM), and cardiovascular disease, which suggests a role of orexins in metabolic homeostasis.^8^ ^,^^9^ It is separately reported that metabolic alterations in persons with type 1 narcolepsy are BMI-independent, implicating orexin physiology in insulin resistance.^10^

The effects of orexins on sleep–wake imbalance, reward and cognitive functions provided the impetus to ascertain whether orexin antagonists are effective at ameliorating depressive symptoms in adults with major depressive disorder (MDD), wherein alterations in orexin signaling have also been reported.^11^ ^,^^12^ Seltorexant, a selective orexin 2 receptor antagonist (SORA) in late-phase development as an adjunctive treatment in MDD, has demonstrated significant antidepressant effects especially in persons with moderate to severe baseline levels of insomnia [ie, Insomnia Severity Index (ISI) ≥ 15].^13^ ^,^^14^ The average effect size estimate of the antidepressant effect of seltorexant is also noted to be meaningful in persons with low severity of insomnia, implicating other mechanistic bases for its antidepressant action.^14^ The differential receptor selectivity of DORAs (both OX1Rs and OX2Rs) compared to the SORA (predominantly OX2R) may have distinct implications for arousal, stress responsivity and metabolic regulation. However, the mechanistic substrates subserving the antidepressant effects observed with dual orexin antagonists (DORAs) and more convincingly with the single orexin antagonist (SORA), seltorexant, are not well characterized.

Multiple non-monoaminergic targets are implicated in depressive symptom alleviation in MDD, including, but not limited to, glutamatergic, GABAergic, and sigma-1.^15^ ^,^^16^ Convergent evidence also implicates metabolic disturbances in the pathophysiology of depression, suggesting that the mechanistic basis of some conventional and/or investigational antidepressants may involve effects on metabolic systems.^17^ For example, molecular crosstalk between N-methyl-D-aspartate (NMDA), incretin, and insulin receptors may be a pharmacodynamic point of convergence for ketamine, esketamine, and the preliminary evidence for incretin receptor agonists (ie, GLP-1 receptor agonists) in the treatment and prevention of depressive symptoms.^18^ ^–^^20^

The aforementioned findings implicating orexins in MDD, along with the observed effects of orexins on metabolic systems, introduce a viable and testable hypothesis that the antidepressant effects of orexin antagonism may be partially mediated by their effects on metabolic parameters (eg, glucose–insulin homeostasis). Herein, we synthesize results from preclinical, pharmacologic, and clinical studies that have reported on the effects of orexin, as well as DORAs and SORAs, on glucose–insulin homeostasis. The overarching aim is to empirically support a proposed mechanistic model implicating metabolic signaling modulation as relevant to the antidepressant effect of orexin targeting. In accordance with our overarching aim, we introduce a model that is preliminary and awaits further evidence to establish and refine the conceptual framework. A comprehensive framework regarding the pathogenesis of depression cannot be explained by a singular effector; instead, we highlight the orexin system to encourage scrutiny of this particular effector without the intention of suggesting that the orexin system itself would be adequately explanatory.

## Methods

This review is a narrative, hypothesis-driven perspective intended to highlight recent translational advances and propose a novel mechanistic framework linking orexin modulation, glucose–insulin homeostasis, and antidepressant effects. As comprehensive studies of endogenous orexin signaling on glucose–insulin homeostasis have been previously published,^2^ ^,^^21^ we do not aim to provide a comprehensive or systematic review of the literature.

A search was conducted on PubMed for studies published from inception to June 2025. Search terms included combinations of keywords related to orexin, orexin antagonists, glucose, insulin, metabolism, and depression. Additional relevant articles were identified through citation searching. The search yielded approximately 158 records.

No formal eligibility criteria were applied, as we aimed only to highlight representative and conceptually informative findings of the proposed framework. Eligible studies included primary preclinical and clinical studies reporting on the effects of orexin signaling and modulation on factors associated with glucose–insulin homeostasis, including energy expenditure, adipose tissue, gluconeogenesis, and sleep-related effects on insulin resistance. The identified records were assessed for relevance to the intersection of orexin signaling and metabolic processes, with a particular focus on implications for depressive phenotypes. Articles were prioritized if they: (1) evaluated the effects of orexin modulation on glucose–insulin homeostasis, (2) investigated biological mechanisms through which orexins may influence metabolic function, and (3) provided translational or clinical insight relevant to depression.

Results were synthesized qualitatively to integrate evidence across experimental levels and to inform the proposed mechanistic model. No formal quality appraisal or quantitative analysis was performed. Therefore, the resulting synthesis of the findings is presented narratively and proposes key mechanisms by which orexins may modulate metabolic function. In cases where multiple studies assessed similar outcomes, representative or conceptually informative examples were selected to illustrate concepts relevant to the proposed framework.

## Results

Evidence implicating the governance role of orexins in metabolism is synthesized in Table 1. Converging evidence suggests that orexin signaling coordinates multiple metabolic functions, incorporating energy sensing with gluconeogenesis and sleep–wake homeostasis. However, some inconsistencies related to energy expenditure underscore the context-dependent effects of orexin activity on metabolism.

**Table 1.** Postulated mechanisms wherein orexin signaling affects metabolic processes Table 1. long description.

The endocrine pancreas produces orexins and expresses orexin receptors
Orexins govern energy expenditure
Orexins influence gluconeogenic activity
Orexins modulate sleep architecture (ie, REM and non-REM sleep)

### Orexin neurons, receptors, and effects on other metabolic peptides: Glucose sensors and central hypothalamic metabolic regulators

The tissue distribution of orexin receptors is well-characterized, both within the central nervous system and in the periphery.^21^ ^,^^22^ Notably, OX1Rs are present in the hippocampus, locus coeruleus, and raphe nuclei.^22^ Orexin-containing neurons act as metabolic biosensors within the LH/PFA and are activated by stress, glutamate, nicotine, GLP-1, corticosterone, and hypoglycemia.^2^ ^,^^23^ ^,^^24^ In accordance with the foregoing, preliminary evidence indicates that central administration of an orexin antagonist (ie, OX1R antagonist) reduced olanzapine-induced hyperglycemia, which was hypothesized to be due to blockade of olanzapine-induced stimulation of orexin-containing neurons in the LH/PFA.^25^

Peripherally, orexin receptors are located in the adrenal gland, the myenteric plexus of the gastrointestinal tract, as well as in the endocrine and exocrine pancreas.^21^ With respect to the direct effects of orexin on insulin signaling, orexin receptors, primarily OX1Rs, are expressed on the pancreatic alpha and beta cells.^26^ Notably, the production of orexin 1 by the endocrine pancreas appears to be higher than that of orexin 2.^2^ Furthermore, pancreatic islet production of orexin is reported to stimulate insulin secretion and decrease glucagon, suggesting an incretin-like role.^27^ Emerging evidence also indicates that circulating plasma orexin levels are negatively associated with measures of insulin resistance, further supporting the role of orexins in modulating insulin sensitivity.^28^ Notably, orexin signaling involves both OX1Rs and OX2Rs, both of which are implicated in metabolic processes. The disproportionate expression of OX1Rs in pancreatic islets suggests that their function and possibly pharmacologic modulation may not have effects identical to those of orexin 2 modulation. The metabolic effects of separately modulating OX1Rs and OX2Rs are a priority for future research and require further characterization.

The effect of orexins on metabolic systems may also be indirect through the modulation of other metabolism-related peptides. For example, adiponectin, an adipokine produced by peripheral and subcutaneous adipose tissue, is known to have insulin-sensitizing, anti-inflammatory, and anti-atherogenic effects.^29^ Orexin 1 is known to stimulate the secretion of adiponectin from adipocytes via PPAR-γ-mediated signaling.^30^ The complex role of orexins in metabolism is underscored by their glucose-sensing effects. For example, it is estimated that approximately 50% of orexin neurons, notably in the PFA, are critical for hypoglycemia awareness and are triggered by hypoglycemia, as well as by ATP and lactate, acting as energy sensors.^31^ ^–^^34^ Taken together, convergent central and peripheral mechanisms underscore the role of the orexin system as a bidirectional regulator of metabolic homeostasis, neuroendocrine signaling, and neuropsychiatric function. These observations suggest that orexin-mediated modulation of insulin and incretin signaling may influence depressive symptoms known to be affected by metabolic functions (eg, reward and cognitive function, and stress responses).

### Effect of orexins on energy expenditure and thermogenesis

Orexins are also known to affect locomotor activity, suggesting that activity-related energy expenditure may contribute to changes in overall energy expenditure.^35^ ^,^^36^ Moreover, exercise is reported to increase plasma orexin 1 levels, consequently increasing sympathetic nervous system activity and energy expenditure.^37^ Functionally, orexins are also known to affect multiple systems that increase energy expenditure at the cellular level.^35^ ^,^^38^ Replicated evidence indicates that orexins govern non-exercise–induced thermogenesis and energy balance.^37^

For example, orexin receptors are expressed on human adipose tissue.^39^ Brown adipose tissue is known to regulate thermogenesis and energy expenditure, and its induction reduces obesity and weight gain.^40^ Central infusion of orexins has been demonstrated to increase the proportion of brown-to-white adipose tissue.^40^ Orexins (especially orexin 1) also facilitate an increase in thermogenesis in brown adipose tissue along with promoting lipolysis in white adipose tissue.^41^ Specifically, orexin 1 has been reported to contribute to the development and maintenance of thermogenic activity.^42^ ^,^^43^ In addition, orexin 1 and orexin 2 have been reported to modulate PPAR-γ2 mRNA expression, which indicates a direct effect of orexins on lipid metabolism and adipogenesis.^39^ Finally, orexins affect preadipocyte proliferation, lipolysis, glucose uptake, lipogenesis, and leptin mRNA expression. Taken together, the aforementioned collection of findings implicates orexins as moderators of brown adipose tissue morphology. These effects may have downstream relevance for depression phenotypes, especially in individuals with alterations in motivation and reward processing.

### Orexin modulation influences gluconeogenic activity

The mechanisms that subserve the beneficial effects of orexin receptor antagonists on glucose–insulin homeostasis are detailed in Table 2. Across studies, orexin antagonism demonstrates convergent effects on neuroendocrine activity and metabolic regulation. However, whether these improvements reflect direct metabolic actions or are secondary to sleep normalization remains unclear. The liver maintains glucose homeostasis by balancing glucose uptake with hepatic glucose production, a process governed by the autonomic nervous system and the suprachiasmatic nuclei (SCN).^23^ Neuronal projections from the SCN to the orexin-containing neurons increase orexin secretion and exert effects on peripheral glucose homeostasis.^23^ Preclinical evidence demonstrates that suvorexant administration in type 2 diabetic *db/db* mice reduces mRNA expression levels of hepatic gluconeogenic factors, phosphoenolpyruvate carboxykinase (Pepck) and peroxisome proliferator-activated receptor-gamma coactivator-1 alpha (Pgc-1alpha), and glucose-6-phosphatase (G6Pase).^44^ In addition, phosphorylated CREB, known to promote gluconeogenic genes, was also significantly reduced.^44^

**Table 2.** Beneficial effects of orexin antagonism on glucose–insulin homeostasis: mechanisms Table 2. long description.

• Normalization of REM and non-REM sleep
• Reductions in sympathoadrenal overactivity
• Reducing cortisol homeostatic disturbances
• Reduces gluconeogenesis

With respect to clinical evidence, suvorexant administration is also reported to decrease serum glucose, independent of its effects on body weight and BMI, implicating reductions in sympathoadrenal activity.^45^ Emerging trends also suggest that orexin receptor antagonists may reduce cortisol homeostatic disturbances, introducing another mechanism through which they can improve glucose–insulin homeostasis.^46^ ^,^^47^ It has been reported that suvorexant administration in type 2 diabetic *db/db* mice also improved impaired glucose tolerance without affecting systemic insulin sensitivity or serum insulin.^44^ Separately, lemborexant administration in type 2 diabetic *db/db* mice showed an increase in REM and non-REM sleep time followed by improved glucose tolerance without enhancing insulin secretion in *db/db* mice.^48^

Consistently, suvorexant administration in humans with T2DM improves glycemic control as measured by continuous glucose monitoring.^49^ In addition, suvorexant may decrease hyperglycemia related to the dawn phenomenon, suggesting a suppression of sympathetic nerve activity as a consequence of improved sleep quality.^49^ ^,^^50^ Overall, the foregoing evidence suggests that pharmacologic orexin antagonism may indirectly have clinically meaningful effects on insulin sensitivity. Notably, glucose–insulin dysregulation is associated with attenuated antidepressant response, suggesting that metabolic modulation may partially mediate the effects of orexin antagonists.

### Orexins modulate sleep architecture

Sleep behavior and architecture are known to affect glucose–insulin homeostasis. A meta-analysis of 245 observational studies reported that the presence of a sleep disorder is associated with a higher rate of T2DM (ie, 39.7%, 95% CI = [34.9, 44.7]) than the presence of any other mental disorder.^51^ Moreover, sleep disturbance in individuals living with MDD is a potent prognosticator of subsequent obesity.^52^ ^,^^53^ Separately, it has been reported that REM sleep was associated with more stable glucose levels while non-REM sleep was associated with increased glucose levels when individuals underwent a glucose tolerance test.^54^ In addition, regulation of circadian rhythms by the orexin system has a bidirectional relationship with glucose control,^23^ suggesting that modulation of sleep architecture may represent an indirect mechanism through which the orexin system exerts its effects on metabolism and metabolic function. While sleep disturbances are well-established in depression populations, it is a current research imperative to delineate sleep-mediated improvements in mood from direct antidepressant effects of orexin antagonists.

## Discussion

A strategic imperative in mood disorder therapeutics is to identify mechanistically novel agents that improve upon clinical outcomes achieved with conventional therapies. Ketamine and esketamine are the most transformative treatments in the treatment of depressive disorders in almost seven decades, as evidenced by their efficacy in difficult-to-treat depression, rapid onset of action, and reduction in measures of suicidality.^55^ In addition, GABAergic modulators (eg, zuranolone) represent innovation insofar as they target the excitatory–inhibitory imbalance and provide rapid symptom improvement in postpartum depression.^56^ The FDA approval of dextromethorphan–bupropion represents the first oral, rapid-acting NMDA, sigma-1 receptor antagonist combination with clinically meaningful effects on measures of anhedonia.^16^ The foregoing findings demonstrate the therapeutic potential of targeting alternative neurobiological systems involved in synaptic plasticity and network regulation, as well as investigating integrative systems.

Multiple lines of evidence implicate disruptions in metabolic function in the pathophysiology of mood disorders. In addition, impairment in insulin signaling reduces the probability of remission reported with conventional psychotropic treatments for mood disorders.^57^ ^,^^58^ Robust preclinical, translational, and preliminary clinical evidence accords with a hypothesis that CNS-penetrant incretin receptor agonists could be potentially disease-modifying in persons with mood disorders.^18^ ^,^^19^ Orexins may represent a convergent interface between neurobiological and metabolic processes, providing a plausible, non-mutually exclusive mechanistic framework for depression pathophysiology.

Several intersecting biological systems provide context for the involvement of orexin signaling in depression. Metabolic dysregulation is associated with systemic inflammation, which is implicated in depression pathophysiology. Orexin signaling modulates cytokine activity, cortisol dynamics, and stress responses, suggesting a contributory immunometabolic component. Moreover, orexin neurons project to mesolimbic and mesocortical circuits involved in reward processing and motivational behavior, which are commonly disrupted in depression. Insulin signaling influences reward salience and decision-making, underscoring a plausible orexin-mediated integration of metabolic signaling that may contribute to depressive symptomatology.

Orexin antagonists are mechanistically informed therapeutics FDA-approved for sleep–wake imbalance, with efficacy in the acute and long-term treatment of insomnia. The commercially available DORAs that are FDA-approved are known to improve behavioural measures of sleep quality, quantity, and efficiency without evidence of cognitive impairment, respiratory suppression, motor vehicle driving impairment, or rebound effects. The SORA, seltorexant, is in Phase 3 development as an adjunctive treatment in adults with MDD. The rationale for developing a SORA, rather than a DORA, is provided by preclinical and pharmacologic evidence indicating that the OX2R has differentiated effects on brain substrates implicated in the stress response.

Alternative mechanisms may account for the observed effects of orexin antagonists. Improvements in sleep architecture may independently drive mood improvements, while modulation of circadian biology may represent an additional pathway distinct from metabolic effects. Orexin antagonists likely affect multiple effectors, requiring further investigation to disaggregate contributory pathways.

Orexins and orexin receptor antagonism affect glucose–insulin homeostasis, glucose tolerance, adipose tissue morphology, and thermogenesis, as well as augmenting effects on other systems integral to metabolism (eg, adiponectin). Preliminary results from animal models, pharmacologic and clinical studies indicate that orexin antagonism has clinically meaningful effects on metabolic parameters. From a clinical perspective, the metabolic effects are welcomed in light of the high rate of metabolic disturbance in persons with depressive and bipolar disorders, especially among those who are treatment-resistant.^59^ ^,^^60^ Conceptually, it is a viable hypothesis open for testing that the antidepressant effects of seltorexant (and perhaps other orexin modulators) may be partially mediated by effects on metabolic systems. A related and separate hypothesis would be that the benefit of orexin modulation, if partially mediated by metabolic systems, would be especially pronounced in measures of anhedonia, reward-based decision-making and cognition.

A limitation of this review derives from our method insofar as it is not systematic. We recognize that there is an incomplete database with respect to characterizing the effects of orexins on metabolic measures, especially in the psychiatric population. The evidence for the antidepressant efficacy of orexin modulators remains limited, with available studies being heterogeneous in design. The inferences regarding the relationship between orexin, metabolism, and depression are plausible, but are based on preclinical, preliminary evidence and have not yet been validated clinically. Both orexin and metabolic processes are complex and affected by multiple factors. It is difficult to isolate the contribution of their effectors. Separately, the commercially available DORAs have not yet documented antidepressant effects in large, adequately powered randomized controlled studies, and hitherto, only the SORA, seltorexant, has demonstrated antidepressant effects as part of large, adequately powered randomized controlled studies. Results from late-phase studies with seltorexant are expected soon; whether its antidepressant effects are partially mediated by benefits on glucose–insulin homeostasis would be informative for further discovery and development activities and provide insights into its potential mechanism of action. Notwithstanding the aforementioned limitations, it remains a testable hypothesis as to whether modulation of the orexin system leads to clinically meaningful improvements in depressive symptom severity and whether these effects are partially mediated by metabolic systems.

## Long descriptions

### Table 1. Long description

The table consists of a header row followed by three body rows, each containing a bulleted point.

* Header row: The endocrine pancreas produces orexins and expresses orexin receptors.

* Second row: Orexins govern energy expenditure.

* Third row: Orexins influence gluconeogenic activity.

* Fourth row: Orexins modulate sleep architecture, specifically R E M and non-R E M sleep.

Navigate back to Table 1..

### Table 2. Long description

The table consists of a header followed by three rows of text.

* Header: Normalization of R E M and non-R E M sleep.

* Row 1: Reductions in sympathoadrenal overactivity.

* Row 2: Reducing cortisol homeostatic disturbances.

* Row 3: Reduces gluconeogenesis.

Navigate back to Table 2..
